# An edge sensitivity based gradient attack on graph isomorphic networks for graph classification problems

**DOI:** 10.1038/s41598-025-97956-7

**Published:** 2025-04-29

**Authors:** Srinitish Srinivasan, Chandraumakantham OmKumar

**Affiliations:** School of Computer Science and Engineering, Vellore Institute of Technology, Chennai, India

**Keywords:** Drug discovery, Drug safety

## Abstract

Graph Neural Networks have gained popularity over the past few years. Their ability to model relationships between entities of the same and different kind, represent molecules, model flow etc. have made them a go to tool for researchers. However, owing to the abstract nature of graphs, there exists no ideal transformation to represent nodes and edges in the euclidean space. Moreover, GNNs are highly susceptible to adversarial attacks. However, a gradient based attack based on latent space embeddings does not exist in the GNN literature. Such attacks, classified as white box attacks, tamper with latent space representation of graphs without creating any noticeable difference in the overall distribution. Developing and testing GNN models based on such attacks on graph classification tasks would enable researchers to understand and develop stronger and more robust classification systems. Further, adversarial attack tests in the GNN literature have been performed on weaker, less representative neural network architectures. In order to tackle these gaps in literature, we propose a white box gradient based attack developed from contrastive latent space representations. Further, we develop a strong base(victim) learning spectral and spatial properties of graphs with consideration of isomorphic properties. We experimentally validate this model on 4 benchmark datasets in the molecular property prediction literature where our model outperformed over 75% of all LLM-based architectures. On attacking this model with our proposed adversarial attack strategy, the overall performance drops at an average of 25% thereby clearing a few gaps in the existent literature. The code for our paper can be found at https://github.com/Deceptrax123/An-edge-sensitivity-based-gradient-attack-on-GIN-for-inductive-problems

## Introduction

Graph classification is a fundamental task in machine learning with applications in bioinformatics, social network analysis, and chemistry. Traditional graph neural networks (GNNs) often struggle with distinguishing non-isomorphic graphs due to their reliance on message passing, which can lead to over-smoothing and loss of structural information^1,2^. To address this limitation, Graph Isomorphic Networks (GINs) were introduced, offering a more expressive architecture by leveraging injective aggregation functions^3,4^. GINs have demonstrated theoretical guarantees in capturing graph structures, making them more effective than traditional GNNs in distinguishing complex topologies^5^. Recent advancements have further improved GINs by integrating spectral methods, attention mechanisms, and hierarchical pooling, which enhance their representational power and generalization capabilities across diverse graph domains^6,7^.

Despite their effectiveness, GINs and other GNN variants remain vulnerable to adversarial attacks, where carefully crafted perturbations can significantly degrade model performance^8,9^. These attacks exploit the reliance of GNNs on structural and feature-based dependencies, leading to manipulated graph representations that mislead classification outcomes^10^. Adversarial robustness in GNNs has gained increasing attention, with researchers exploring defensive strategies such as adversarial training, graph purification, and robust message-passing schemes^11–13^. Understanding these vulnerabilities and developing resilient architectures is crucial for deploying GNNs in safety-critical applications.

Therefore, building a graph network that is robust to adversarial attacks is the need of the hour. Another key property in the field of graph theory is graph isomorphism. An isomorphism between 2 graphs is the bijection between their vertex sets preserving adjacency. In an automorphic network, if two nodes correspond to each other, standard graph neural network operations such as GCN will provide similar embeddings to both the nodes^14^. However, different neighborhoods to each of the nodes can be considered giving it different depths in the Breadth First Search(BFS) tree thereby improving the expressiveness of the network. In consideration of all these shortcomings i.e. the vulnerability of graph neural networks to adversarial attacks, the inefficiency of Large Language Models in molecular property prediction and graph isomorphism, we propose a novel technique that makes use of contrastive pre-training and negative sampling to pre-train a spectral graph convolution backbone thus making the network robust to adversarial attacks. Further, we construct a graph isomorphic network with dual path embedding aggregation with the pre-trained spectral backbone to increase the expressiveness of the model. The proposed model achieves an average AUC of 85% outperforming over 97% of all benchmark models with a size of only 835,000 parameters providing an efficiency of over 95% when compared with all benchmark models on the MoleculeNet^15^ datasets. Through our proposed model, we achieve state of the art results and thereby show that graph neural networks are not only expressive but considerably more efficient and robust, thus advancing further research in the domain of molecular graph representation using neural network generated embeddings and drug discovery.

Gradient-based white-box attacks on Graph Neural Networks (GNNs) represent a significant security concern within the domain of deep learning on graph-structured data. These attacks exploit the model’s accessibility by manipulating gradients—calculated directly from the GNN’s parameters—to generate adversarial perturbations. In a white-box setting, attackers have full knowledge of the model architecture, parameters, and gradients, enabling them to optimize perturbations with high precision. By subtly altering node features or graph structure, attackers can mislead the GNN into producing incorrect predictions without noticeable changes to the human eye. This vulnerability is particularly alarming for applications that rely on GNNs, such as social network analysis, molecular property prediction, and recommender systems, where even slight inaccuracies can lead to adverse outcomes.

The serious threat posed by these gradient-based white-box attacks lies in their ability to destabilize systems by degrading model performance in critical scenarios. Since GNNs are increasingly deployed in sensitive and high-stakes environments, adversarial attacks could disrupt financial networks, mislead recommendation algorithms, or compromise cybersecurity applications. Furthermore, due to the interconnected nature of graph data, even minimal perturbations can propagate through the network, compounding the attack’s impact. The accessibility of gradients in white-box attacks allows adversaries to exploit the model’s exact weaknesses, significantly reducing the robustness of GNNs and raising critical concerns about their deployment in real-world applications. Addressing these vulnerabilities requires robust defenses that can detect and counteract these attacks without compromising model performance or interpretability.

## Main contributions

- We present an efficient graph based model that comprises of only 835,000 parameters. The approach makes use of the expressive power of molecular graphs and comprises of very few layers(3) thus making it light-weight with considerably reduced training and inference time. Our proposed approach effectively models the drugs and predicts the side effects on various MoleculeNET datasets by achieving an average AUC score of 85%. Despite the lesser number of parameters, our proposed model produces state of the art results on all prediction tasks.
- A contrastive approach to self-supervised learning by generating reconstructed molecular graphs on original and perturbed embeddings from the latent space. Such a design trains the model to defend itself against adversarial attacks which is a necessary requirement since a small, insignificant feature or weight perturbation may change prediction of the model whose effects can be expensive both clinically and financially.
- We present a novel dual branched graph based model that comprises of a spectral branch, which is pre-trained through the adversarial approach, and an isomorphic branch. Such an approach embeds spatial, spectral and isomorphic features of automorphic graphs thereby generating different embeddings for adjacent nodes of an automorphic molecular graph. Through these techniques, we not only achieve state of the art results when compared against benchmark models but also create an efficient model with less than 1 million parameters.
- We present a novel gradient based attack strategy based on the contrastive embeddings learning during self supervised learning. We generate perturbed graphs by reconstructing the attacked latent space distribution. During inference, the performance of the model drops by an average of 20% on classification datasets and regression(Liphophilicity).

### Literature review

The traditional methods for molecular property prediction prove inefficient and irreproducible making it necessary for using Deep Learning^16^approaches to compensate for a more efficient solution^17^. implemented a pre-trained variational autoencoder for improving the performance of classification tasks^18^. uses a pre-trained BERT model, to generate molecular sentences from compound structures. This is done using the Extended-Connectivity Fingerprint (ECFP) algorithm to create substructure identifiers, which is then processed by NLP^19^. However the Deep Learning methods limit the inputs to the training dataset which is not viable for the complexity and variation present in the molecular characteristics, making it difficult to use these Deep Learning methods on different problem statements. A better performing alternative are graph based algorithms as the node based representations prevent loss of characteristic data, taking more variables into account and hence performing better. Graph Neural Networks^20^implemented with transfer learning^21^perform significantly better because of its ability to use low-fidelity measurements as proxies for high-fidelity properties, allowing for effective learning even when high-quality data is scarce. Integrating Graph Neural Network with representational learning^22^would help in solving the issue of lack of unlabelled data as seen commonly in molecular property prediction. It also helps in generalizing the model to extend it to be implemented wherever required. It is able to learn representations with underlying chemical properties^23^ as opposed to other methods. Table 1further contains the related work for our study^48^. introduces a simplified version of PCNet (SPCNet) that enhances robustness against graph structure perturbations and adversarial attacks. It extends the filter order to continuous values, reduces parameters, and implements variants with adaptive neighborhood sizes^49^. addresses semantic heterophily in unsupervised heterogeneous graph representation learning (UHGRL) by proposing LatGRL. LatGRL constructs fine-grained homophilic and heterophilic latent graphs using structure/attribute similarity mining to guide representation learning. An adaptive dual-frequency semantic fusion mechanism and scalable implementation further enhance its effectiveness and efficiency, validated on benchmarks.

**Table 1:** Literature survey on adversarial attacks.

Reference	Summary
^24^ Zügner, D., & Günnemann, S. (2019). Adversarial Attacks on Graph Neural Networks via Meta Learning.	In this foundational work, Zügner and Günnemann introduce a method to perturb both graph structure and node features, effectively bypassing model defenses. They leverage meta-gradients to train adversaries, highlighting vulnerabilities in common GNN architectures, including Graph Convolutional Networks (GCNs). This study is pivotal in demonstrating the power of structural attacks on GNN models, setting the stage for further research into both adversarial techniques and defense mechanisms.
^25^ Dai, H., Li, H., Tian, T., Huang, X., Wang, L., Zhu, J., & Song, L. (2018). Adversarial Attack on Graph Structured Data.	Dai et al. propose a reinforcement learning-based attack framework that operates in an evasion attack setting. This work is significant for introducing a flexible model that learns effective perturbations on graphs, challenging GNNs in tasks like node classification and link prediction. Their method underscores the adaptability of adversarial attacks across various graph-based applications.
^26^ Xu, H., Chen, Y., Liu, S., Chen, P. Y., Weng, T. W., Hong, M., & Lin, X. (2019). Topology Attack and Defense for Graph Neural Networks: An Optimization Perspective.	Xu et al. frame adversarial attacks as an optimization problem and examine methods to alter graph topologies with minimal changes. This study contributes to the understanding of how minor structural perturbations can induce substantial inaccuracies in GNN predictions, offering insights into the creation of more robust defenses.
^27^ Bojchevski, A., & Günnemann, S. (2019). Adversarial Attacks on Node Embeddings via Graph Poisoning.	Bojchevski and Günnemann analyze poisoning attacks on node embedding models, focusing on altering graph structure during the training phase. Their approach effectively reduces the accuracy of GNNs, particularly in node classification tasks. This study is critical for understanding the impact of adversarial manipulation on node embeddings in large networks.
^28^ Wu, L., Zhu, Z., Cheng, H., & He, X. (2019). Adversarial Examples and Model Robustness for Deep Learning on Graphs.	Wu et al. explore adversarial examples in graph data, providing empirical evidence on the vulnerability of GCNs to structural perturbations. They further propose heuristic defenses against these attacks, such as feature smoothing. This work is essential for its early recognition of GNNs’ sensitivity to adversarial examples and for stimulating the development of robust GNN architectures.
^29^ Sun, L., Hong, Y., Yang, X., & Liu, Z. (2020). Adversarial Attacks on Graph Neural Networks in Node Classification: A Hierarchical Reinforcement Learning Approach.	Sun et al. introduce a hierarchical reinforcement learning approach for executing node classification attacks on GNNs. Their method adapts to varying levels of attack granularity, showcasing its efficacy in diverse graph structures. This work expands the attack framework beyond simple perturbations, emphasizing the hierarchical nature of certain graph-based adversarial threats.
^30^ Wang, X., Han, Y., Zhao, X., & Li, L. (2020). Certified Robustness for Graph Neural Networks against Adversarial Attacks.	Wang et al. develop a certification framework to measure the robustness of GNNs against adversarial perturbations. This approach is among the first to offer formal guarantees for GNN robustness, using robust training methods to counteract adversarial influence. Their findings highlight that robustness can be systematically quantified and enhanced in GNNs.
^31^ Chen, Z., Li, X., Wu, L., & He, X. (2020). GNNGuard: Defending Graph Neural Networks against Adversarial Attacks.	This study introduces GNNGuard, a defense mechanism that dynamically assesses the reliability of a node’s neighborhood in response to adversarial perturbations. By filtering potentially adversarial neighbors, GNNGuard increases the resilience of GNNs against various attacks. This technique contributes a practical solution for enhancing GNN security in adversarial settings.
^32^ Jin, W., Ma, Y., Liu, X., Tang, X., & Wang, J. (2021). Node Similarity Preserving Adversarial Attack for Graph Neural Networks.	Jin et al. focus on preserving node similarity while crafting adversarial attacks, allowing perturbations to remain undetected. This novel approach explores the trade-off between attack efficacy and stealth, addressing practical adversarial scenarios where perturbations must maintain the original graph’s structural integrity.
^33^ Chen, Z., Wu, L., & He, X. (2021). Topology-Aware Adversarial Attack and Defense for Graph Neural Networks.	Chen et al. propose a topology-aware approach that emphasizes attacking and defending based on the inherent topology of graphs. Their method effectively demonstrates that topology-aware defenses offer a significant increase in robustness, reinforcing the need for defenses that account for structural dependencies.
^34^ Zhang, X., Chen, Z., Liu, H., & Han, J. (2022). Adversarial Attack and Defense Strategies for Graph Neural Networks: A Survey.	Zhang et al. present a comprehensive survey that categorizes various attack and defense methodologies in GNNs, discussing their strengths, weaknesses, and applicability across graph-based tasks. This survey consolidates the knowledge in adversarial GNN research, highlighting the critical need for robust, scalable defense mechanisms.

## Methodology

### Initial node vector initialization

**Fig. 1:**
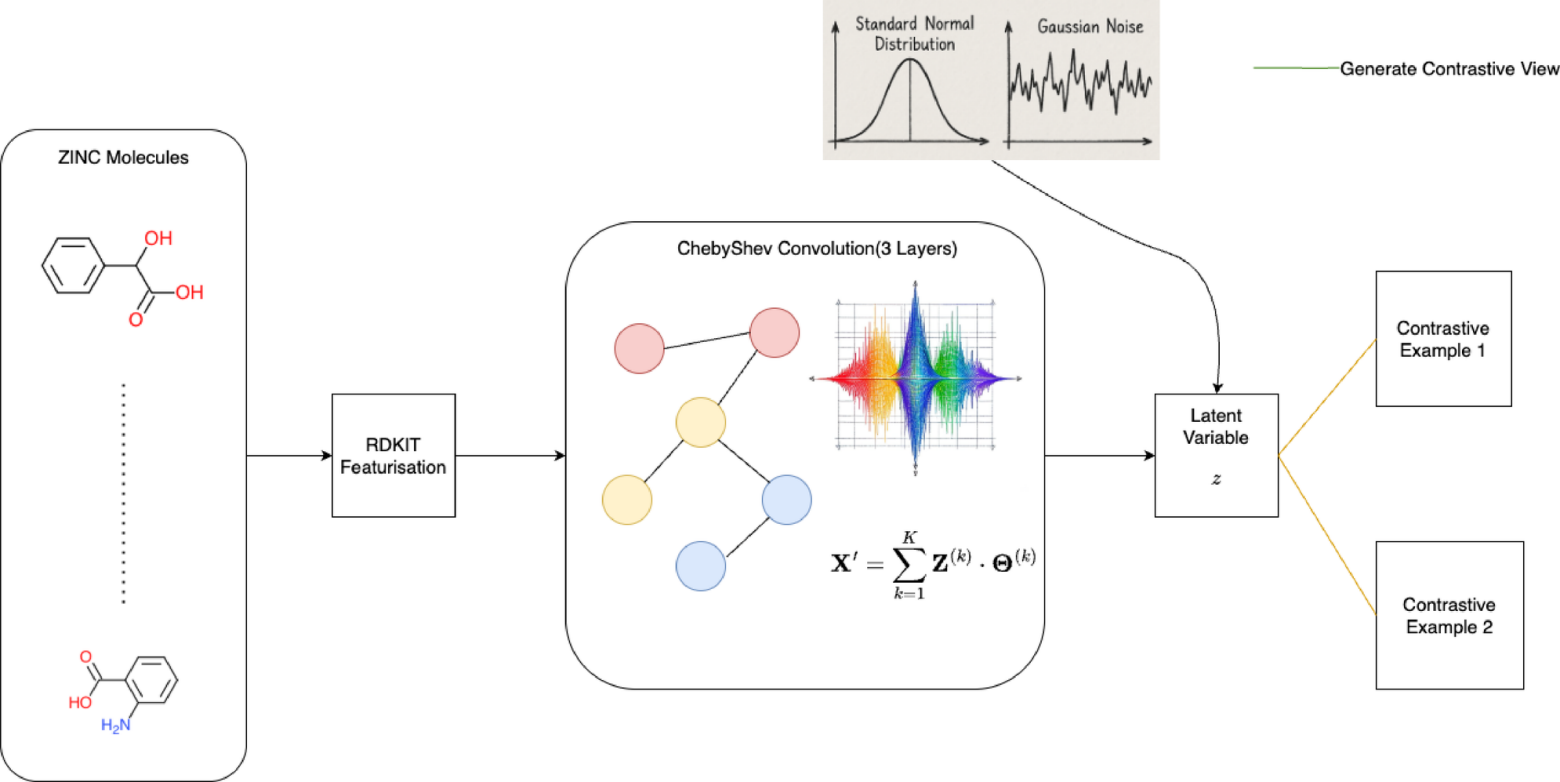
Contrastive Pre-training on ZINC Database.

**Fig. 2:**
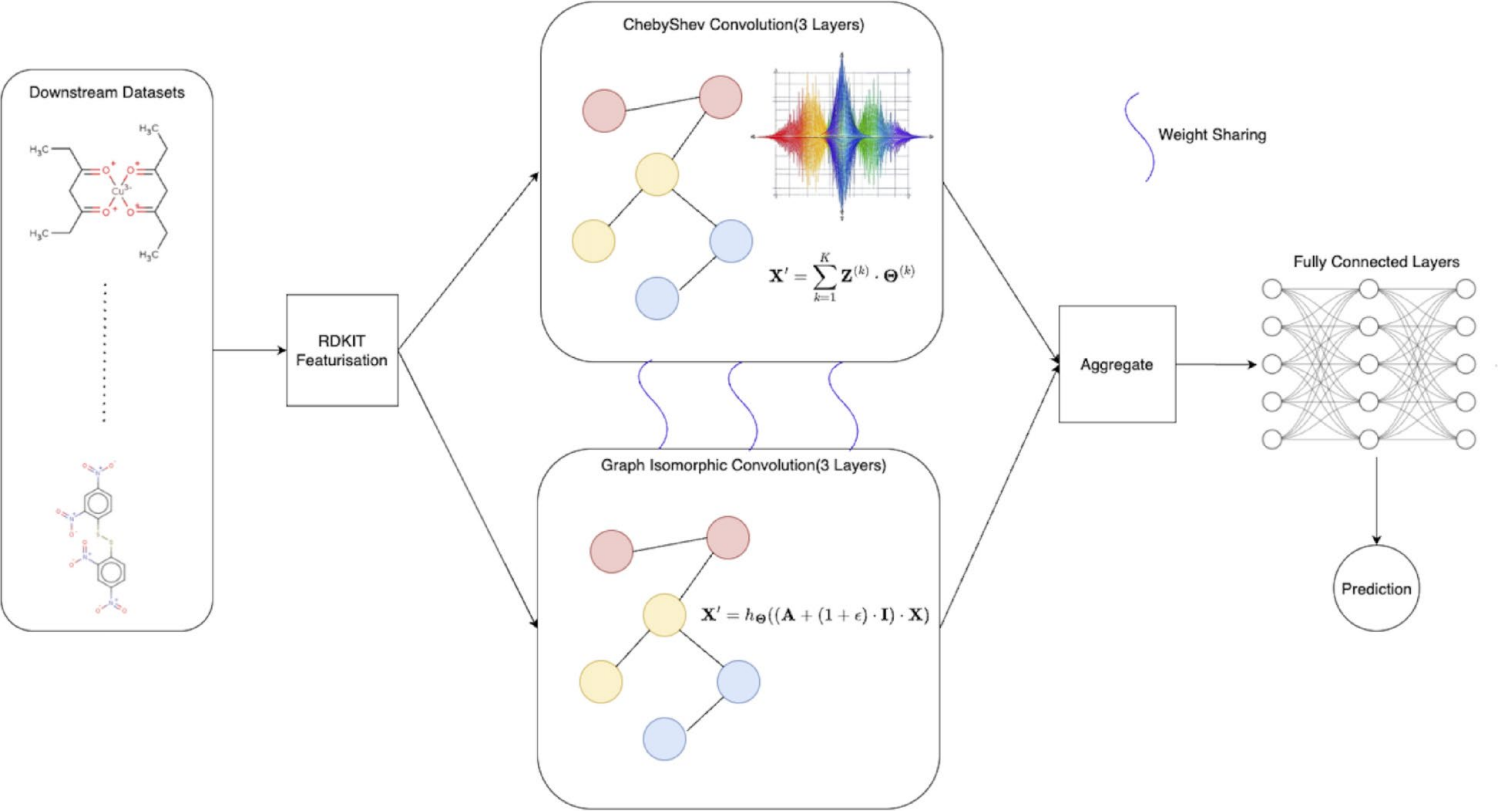
Dual Branched Network for prediction on Downstream Datasets.

 Figures 1 and 2 describes the classification and self supervised stage of our proposed method.

## Graph definition

The Chemical SMILE String of each reactant of the reaction is converted to a Molecular Graph in the Graph Space followed by feature vectors for each atom in the Euclidean Space. An atom is represented as a graph node while a bond is represented as a graph edge. Each node is represented by a 56-dimensional feature vector and each edge is represented by a 9-dimensional vector. Equation 8 depicts the graph definition.

1 $${\rm G = (A,B)}$$

where, G = Molecular Graph.

A = [v1,v2,….,v56].

B = [e1,e2,….e9].

In Eq. 1, A refers to the 56 dimensional embedding vector for each atom of the graph, B refers to the 9 dimensional embedding vector for each bond.

### Node features

On the atomic level, the following features were used to featurise each vertex V of graph G:

- Formal Charge.
- Degree of each Atom.
- Hybridization.
- Presence in Ring.
- Aromaticity.
- Atomic Mass.
- Vander waal’s Radius.
- Covalent radius.

**Formal Charge** refers to the charge carried by each atom in a molecule. Equation 10 describes the computation of formal charge

2 $$\:\text{Formal Charge}=V-N+\frac{B}{2}$$

In (2), V stands for number of valence electrons, N stands for number of non bonding valence electrons and B stands for total number of electrons shared in bonds. The range for formal charge we have considered is from − 3 to 3.

**Degree** refers to the number of neighboring atoms interlinked by bonds. The range of degree we have considered is from 0 to infinity.

**Table 2:** Hybridization type based on the number of hybrid orbitals.

Number of Hybrid Orbitals	Hybridization
2	$\:sp$
3	$\:s{p}^{2}$
4	$\:s{p}^{3}$
5	$\:s{p}^{3}d$
6	$\:s{p}^{3}{d}^{2}$

#### **Hybridization**

refers to the intermixing of atomic orbitals with same energy levels to produce a new type of atomic orbitals of the same number in accordance with the Valence Bond Theory(VBT). The type of hybridization gives the geometry of the compound and bond angle. It is indirectly calculated by computing the number of hybrid orbitals(described in (3)) of the central atom which is mapped to the hybridization type described in Table 2. 

3 $$\:H=\frac{1}{2}(V+M-C+A)$$

where H stands for the number of atomic orbitals of central atom, V stands for Number for Valence Electrons of central atom, M stands for the number of surrounding monovalent atoms, C refers to charge on cation and A refers to the charge on anion.

#### **Presence in ring**

A binary feature(1 or 0) whose value is 1 if the atom is part of a ring, otherwise 0.

**Aromaticity** of a compound refers to a property where cyclalkenes are conjugated. They enhance the stability of a molecule by delocalization of π-π electrons due to formation of more than 1 resonant structures.

**Atomic Mass** is the average relative mass of an atom as compared to an atom of carbon isotope 12 i.e. C-12. We use the scaled form which is computed using (4).

4 $$\:\text{Atomic Mass Scaled}=\frac{\text{Mass of Atom}-10.812}{116.092}$$

**VanderWaal** Radius of an atom refers to half the distance between the center of nuclei of two atoms held together by week vander waal’s force. We use the scaled function which is computed using (5)

5 $$\:\text{VanderWaals Radiu's Scaled} = \frac{{\rm VDWR} -1.5}{0.6}$$

**Covalent Radius** refers to the distance between the center of nuclei of two atoms when bound together by a single covalent bond. We use the scaled form which is computed in (6).

6 $$\:\text{Covalent Radius Scaled}=\frac{\text{CVR}-0.64}{0.76}$$

Where CVR refers to the covalent radius.

All features are computed and aggregated using (7).

7 $$\:\text{Atom Vector}=\text{FC}+\text{D}+\text{HYB}+\text{Ring}+\text{ARO}+\text{AM}+\text{CV}+\text{VDR}$$

where FC refers to Formal Charge, D refers to Degree, HYB refers to the one hot vector for hybridization type, Ring is a binary variable if atom is a part of a ring, ARO refers to aromaticity, CV refers to covalent radius and VDR refers to Vander waal’s radius.

## Edge features

The following bond features were used to featurize the edges E of graph G.

- Bond Type
- Conjugation
- Stereochemical Aspects

**Bond** Type refers to the type of bond between two atoms. It maybe a single, double or triple bond or aromatic.

**Conjugation** is represented by a binary variable(1 or 0). The value is 1 if the bond is a part of a conjugation i.e. alternate single and double bond resulting in delocalization of π-π electrons forming different resonant structures, otherwise its 0. Figure 1: Contrastive Pre-training on ZINC Database.

Figure 2: Dual Branched Network for prediction on Downstream Datasets.

**Stereochemical** refers to the usage of E-Z stereochemical features around double bonds. Equation (8) describes the equation we used to featurize bonds.

8 $$\:\text{Bond Vector}=\text{Bond Type}+\text{Conjugation}+\text{E-Z Features}$$

## Contrastive pre-training based self supervised learning

Owing to the shallowness of the initial node vectors, a pre-training stage for the molecular graphs to learn meaningful embeddings is required. The base model for pre- training the reactant is a Graph Autoencoder^35^. Using the Graph Autoencoder, we develop a contrastive training design through embedding perturbation to develop an encoder that is robust to adversarial attacks. The encoder is then used as the backbone of the model whose pre-trained weights are transferred to a classifier or regression model.

### Design of backbone

In this section, we describe the architecture of the backbone that is to be pre-trained. The encoder contains 3 spectral graph convolution layers^36^. Each layer embeds the feature vector to 128,256 and 512 dimensions respectively. The spatial domain of the graphs are preserved. After each layer, we apply Symmetric Normalization followed by ReLU activation for non-linearity. The Kernel size of each spectral filter is 3. Equations (9)-(12) describe the computation of spectral graph convolutions^36^.

9 to 12 $$\:\begin{array}{c}{X}^{{\prime\:}}={{\Sigma\:}}_{k=1}^{K}{Z}^{k}.{{\Theta\:}}^{k}\\\:\text{w}\text{h}\text{e}\text{r}\text{e}\:{Z}^{k}\text{i}\text{s}\:\;\text{a}\:\text{r}\text{e}\text{c}\text{u}\text{r}\text{s}\text{i}\text{v}\text{e}\:\:\text{v}\text{a}\text{r}\text{i}\text{a}\text{b}\text{l}\text{e}\:\;\text{c}\text{o}\text{m}\text{p}\text{u}\text{t}\text{e}\text{d}\:\;\text{a}\text{s}\:{Z}^{1}=X\\\:{Z}^{2} ={L}.X\\\:{Z}^{3}= 2 .{L}.{Z}^{k-1}-{Z}^{k-2}\end{array}$$

Where ${L}$ stands for the normalized Laplacian Matrix which is computed as per (13)

13 $${L}=\frac{2 L}{{\lambda\:}_{max}}-I$$

The Laplacian Matrix $\:L$ is computed as per (14)

14 $$\:L=D-A$$

where, D is the Diagonal Matrix of degrees of each node and A is the Adjacency Matrix. $\:{\lambda\:}_{max}$ refers to the highest Eigen value of the graph Laplacian.

### Training design

Each molecular graph is passed into the backbone discussed in the previous section to form the embedding vector z in the latent space. The embedding vector z is then perturbed using a Normal Distribution to form vectors z1 and z2. The perturbed vectors z1 and z2 are formed by adding z to unit vectors k1 and k2 which is multiplied with 2 points ϵ1 and ϵ2 sampled from a normal distribution of mean 0 and variance $\:{\sigma\:}^{2}$ which is the variance of the true embedding z. We then reconstruct the graphs from embedding vectors z, z1 and z2 using $\:z{z}^{T}$ for all 3 embeddings followed by sigmoid operation over the formed adjacency matrices to form probability scores for each possible edge. Through this stage we develop 3 graphs where one of the graphs the actual true graph and the other two are invalid. To penalize the formation of such graphs, we use a loss function based on Information Loss which is used as a regularizer term and reconstruction loss which we aim to optimize. The following Eq. 15 to 20 describe the step by step implementation of the formation of perturbed embeddings and reconstruction.

15-16 $$\begin{aligned}{k_1} = \frac{k_{1}}{\mid k_{1}\mid}\\ {k_2} = \frac{k_{2}}{\mid k_{2}\mid}\end{aligned}$$

Where $\:\left|k1\right|,\left|{k}_{2}\right|$ are magnitude of vectors $\:{k}_{1},{k}_{2}$. ${{k}}_{1}, {{k}}_{2}$ are unit vectors.

17-18 $$\begin{array}{c}{z}_{1} = z + {\epsilon}_{1} \dot {k}_{1}\\ \: {z}_{2} = z + {\epsilon}_{2} \dot {{k}_{2}}\end{array}$$

$\:{z}_{1},{z}_{2}$ are perturbed latent space samples based on gaussian noise.

19-20 $$\:\begin{array}{c}{\epsilon}_{1}\sim\:N(0,{\sigma\:}^{2})\\\:{\epsilon}_{2}\sim\:N(0,{\sigma\:}^{2})\end{array}$$

Where $\:{\sigma\:}^{2}$ is the variance of the embedding $\:z$.

### Loss function

In this section, we describe the loss function used for pre-training. Equation 21 describes the loss function used.

Loss = Reconstruction Loss + λInformation Loss (21).

where λ is the penalty strength term. For our study, we set it to a value of 0.4. Equation 22 to 28 describes the computation of the Information Loss.

22-28 $$\:\begin{array}{c}A=z.{z}^{T}\\\:{A}_{1}={z}_{1}.{z}_{1}^{T}\\\:{A}_{2}={z}_{2}.{z}_{2}^{T}\\\:p=\sigma\:\left(A\right)\\\:{p}_{1}=\sigma\:\left({A}_{1}\right)\\\:{p}_{2}=\sigma\:\left({A}_{2}\right)\end{array}$$

where A, A1,A2 are the Adjacency Matrices formed from embeddings z, z1,z2 respectively.σ(z) refers to the Sigmoid function which generates probability scores for the existence or non-existence of a particular edge denoted by p, p1,p2 respectively. For the following equations, let $\:x$ represent the adjacency matrix of the original molecular graph

29 $$\:\begin{array}{c}\text{I}\text{n}\text{f}\text{o}\text{r}\text{m}\text{a}\text{t}\text{i}\text{o}\text{n}\;\text{L}\text{o}\text{s}\text{s}=-{E}_{xy}log\frac{\text{e}\text{x}\text{p}\left(g\right(x,p\left)\right)}{\text{e}\text{x}\text{p}\left(g\right(x,{p}_{1}\left)\right)+\text{e}\text{x}\text{p}\left(g\right(x,{p}_{2}\left)\right)+\epsilon}\end{array}$$

In the above equation, Exy represents the expectation of the generated graph distribution. The function g is a link function. In our study, we use the cosine similarity which is described in 30.

30 $$\:\text{Cosine Similarity}(X,Y)=\frac{x.y}{\left|\right|x\left|\right|\left|\right|y\left|\right|}$$

The Reconstruction Loss is a cross entropy loss function. The edges of the true molecular graphs represented as x are the positive samples while the negative samples are generated through negative sampling thus providing a knowledge base for false edges as well.

### Initialization

The weights of all the layers of both the encoder and decoder are initialized concerning a Normal Distribution. The mean and variance used to sample weights are 0 and 1 respectively. All biases were initialized with zeros.

### Optimizer

We use the Adam^37^ optimizer for pre-training each reactant. The β1 parameter in Adam is changed to 0.5 for more stable training. β2 remains as 0.999. The learning rate is set to 0.0002.

### Classification phase

In order to consider and embed isomorphic graphs, we include a network to embed such graphs. Isomorphic graphs are graphs that have a similar structure. Such graphs are challenging to detect since the task of finding the nodes that are corresponding in both graphs cannot be solved in polynomial time. The isomorphic segment of the classification network generates embeddings which are shared with each layer of the graph convolution classifier. In this section we describe the architecture of our property prediction network, the theory of graph isomorphism and embedding aggregation.

### Architectural design

In this section, we describe the architecture of the classification network. The network has 2 branches, one corresponds to graph isomorphism and the other is the spectral graph convolution network. The weights for the spectral graph convolution network are transferred from the pre-training stage while the graph isomorphism network is randomly initialized. The input featurised molecular graph is the input to the spectral branch as well as the isomorphic branch. The spectral branch has the same structure as the graph backbone described in 3.3.1 to accommodate for a seamless weight transfer. The kernel size of each spectral filter is 3 and symmetric normalization is employed on the generated node embeddings at each layer. The graph isomorphic branch contains 3 layers performing the graph isomorphic convolution operation described in^38^. Each layer has a linear transformation based link function to learn a wide range of features which is described in the following section. For the link function, we also make use of batch normalization and ReLU activation function to learn more complex underlying features. Each layer transforms the feature vector of each node to a higher dimension. The dimensions of embeddings computed for each layer is tuned as 128,256 and 512 features respectively. A link between the spectral branch and isomorphic branch is designed for each layer in order to share learned features between the 2 branches making the network more robust. The embeddings from the 2 branches are then aggregated using + followed by graph embedding generation. The graph embedding is generated using the Global Mean Pool operation considering all node features. Equation 16 describes the computation of the graph embedding.

31 $$\:\text{Mean}\left(X\right)=\frac{1}{X}{{\Sigma\:}}_{{x}_{i}\in\:X}{x}_{i}$$

The generated graph embeddings are then passed into a ReLU activated linear layer with batch normalization since it reduces internal co-variate shift^39^ transforming the dimension of the embeddings to 256. This is followed by a linear transformation of k features where k is the number of properties for the final output that varies based on the datasets used. Equations 32 and 33 describe the computation of the linear transformation and classification using sigmoid. It is to note that the computation graph for gradients during backward pass includes all layers and branches i.e. all layers of the proposed design is trainable.

32-33 $$\:\begin{array}{c}{x}^{{\prime\:}}=x{W}^{T}+b\\\ {y}=\frac{1}{1+\text{e}\text{x}\text{p}(-{x}^{{\prime\:}})}\end{array}$$

Where W represents the weights matrix and b represent the bias.

### Graph isomorphism and link function

As described in Sect. 1, an isomorphism between 2 graphs is the bijection between their vertex sets preserving adjacency. The problem of determining whether 2 graphs are isomorphic is challenging and requires exponential time. In order to represent such graphs, we use the concepts described by^38^ to generate embeddings for isomorphic graphs. The Weisfeiler-Lehman(WL) test is one of the tests used to determine if 2 graphs are isomorphic or not. The WL test produces a canonical form for each graph. If the canonical forms are not equivalent, then the graphs are not isomorphic. However the inverse does not always hold i.e. if the two canonical forms are equivalent, it may or may not be isomorphic. Similarly, if A is a Graph Neural Network model and it maps two graphs G1,G2 to 2 different embeddings, then the two graphs G1,G2 would not produce equal canonical forms in the WL test and will be classified as non-isomorphic graphs. Hence, there is a requirement for an injective link function that generates graph level embeddings considering all node features. Expression 34 describe an injective function.

34 $$\:f\left({x}_{1}\right)=f\left({x}_{2}\right)\Rightarrow{x}_{1}={x}_{2}$$

Therefore, according to the above expression, if the node features generate different embeddings, then the graphs are not isomorphic. The linear mapping is an injective function, hence it has been employed as the link function making the overall architecture more expressive and robust.

### Loss function

The negative log-likelihood function was considered as the objective function for all our experiments in this study. The negative log-likelihood is a popular approach for classification problems since it satisfies Jensen’s inequality for convexity, and is continuous and differentiable at all points, enabling deep learning models to reach an optimal solution quickly. Equation (18) describes the computation of the loss function.

35 $$\:\begin{array}{c}l(x,y)=\text{m}\text{e}\text{a}\text{n}\left(\right[{l}_{1},{l}_{2},\cdots ,{l}_{n}\left]\right)\\\:{l}_{n}=-{y}_{n}log\left({p}_{n}\right)-(1-{y}_{n})log(1-{p}_{n})\end{array}$$

### Optimizer

Adams Optimizer^37^ was used as our principle optimization algorithm. The β1 parameter was set to 0.9 and β2 parameter was set to 0.999. The learning rate used for all experiments is 0.0002. We train all models for up to 1000 epochs enabling it to reach convergence and obtain an optimal solution.

### Adversarial attack

#### Random attacks

At the time of inference, we attack the model at random interval. This simulates the real world where attacks are often a rarity. To achieve this task, we sample random points from a sine wave constrained between 0 and $\:\pi\:$. Since the range of the sine function is between 0 and 1 at the restricted domain, it can be considered as a probability density function. For each step, if the value of the sine function is greater than or equal to 0.5, we initiate the attack, otherwise the model operates under normal conditions as described in the previous sections.

#### Attack design

We create a novel white-box based attack that considers the contrastive latent space samples generated during the pre-training phase. Since the same backbone has been attached to the classifier, we can make use of the weights learnt by the backbone and the contrastive samples to generate gradient based attacks. If $\:z,{z}_{1},{z}_{2}$ are the true and contrastive pre-trained embeddings at the latent space where $\:{z}_{1},{z}_{2}$ is obtained through injecting $\:z$ with Gaussian noise, we compute the gradient of the loss computed in Eq. (21) with respect to the re-constructed adjacency matrices from $\:z,{z}_{1},{z}_{2}$, The gradients obtained are combined and injected onto the original molecular graph adjacency matrix. The following equations describe this process

$$\:\begin{array}{c}A=z.{z}^{T}\\\:{A}_{1}={z}_{1}.{z}_{2}^{T}\\\:{A}_{2}={z}_{2}.{z}_{2}^{T}\\\:L=\text{L}\text{o}\text{s}\text{s}\text{F}\text{u}\text{n}\text{c}\text{t}\text{i}\text{o}\text{n}(A,{A}_{1},{A}_{2})\end{array}$$

Here LossFunction refers to Eq. (21).

$$\:\frac{\delta\:L}{\delta\:{A}^{{\prime\:}}}=\frac{\delta\:L}{\delta\:A}+\frac{\delta\:L}{\delta\:{A}_{1}}+\frac{\delta\:L}{\delta\:{A}_{2}}$$

$$\:{\mu\:}_{g}=\frac{{\Sigma\:}\frac{\delta\:L}{\delta\:{A}^{{\prime\:}}}}{N}$$

If the value of $\:{\mu\:}_{g}$ is greater than $\:\frac{\delta\:L}{\delta\:{A}^{{\prime\:}}}$ at a particular edge, we drop that edge. Through this process, we preserve the positive semi-definite property of adjacency matrices and successfully generate adversarial attacks on the graph structure.

## Results and discussion

### Hardware requirements

All graphs were pre-processed on a 2022 M2 Macbook Pro. The binaries of each graph were divided into 8 folds to set up multiple processes. Upto 8 concurrent processes were created making use of all the 8 cores of the hardware to convert all SMILE Strings to PyTorch Graph Tensors. The total time taken to pre-process and create all the graph tensors was 5 h.

#### Software requirements

All the source code for our study was written in Python 3.12. We used PyTorch as our primary framework for data preparation, pre-processing, model construction, training, and testing. For processing the chemical SMILE strings and converting them to molecular graphs, we use the RdKit library. For the computation of benchmark metrics such as Area under the Receiver- Operator Curve(ROC-AUC), we use the Torchmetrics library for multi-label and multi-class classification problems.

#### Dataset description

**Table 3:** Experimental validation of victim model. Scores are reported between 0 and 1 on taking an average of 5 runs.

S.No	Dataset	Model	Metric	Score	Parameters
**1**	HIV	DVMP	ROC AUC	0.81	104.1 M
		Uni-Mol	ROC AUC	0.81	-
		ChemBert	ROC AUC	0.79	77 M
		MolXPT	ROC AUC	0.78	350 M
		GAL 30B	ROC AUC	0.76	30B
		GAL 120B	ROC AUC	0.74	120B
		GAL 6.7B	ROC AUC	0.72	6.7B
		GAL 125 M	ROC AUC	0.70	125 M
		PretrainGNN	ROC AUC	0.80	-
		AttentiveFP	ROC AUC	0.76	-
		D-MPNN	ROC AUC	0.77	-
		Ours	ROC AUC	0.79	8,35,000
**2**	ClinTox	MolXPT	ROC AUC	0.95	350 M
		Syn-Fusion	ROC AUC	0.95	45 M*
		Uni-Mol	ROC AUC	0.92	-
		SPMM	ROC AUC	0.91	More than 50 M
		D-MPNN	ROC AUC	0.91	-
		ChemRL-GEM	ROC AUC	0.90	-
		GAL 30B	ROC AUC	0.82	30B
		GAL 120B	ROC AUC	0.83	120B
		GAL 6.7B	ROC AUC	0.78	6.7B
		GAL 125 M	ROC AUC	0.52	125 M
		ChemBert	ROC AUC	0.56	77 M
		Grover(Large)	ROC AUC	0.76	More than 100 M
		PretrainGNN	ROC AUC	0.73	-
		AttentiveFP	ROC AUC	0.85	-
		D-MPNN	ROC AUC	0.91	-
		Ours	ROC AUC	0.95	8,35,000
**3**	BBBP	ChemBert	ROC AUC	0.73	77 M
		Grover(Large)	ROC AUC	0.69	More than 100 M
		SPMM	ROC AUC	0.73	More than 50 M
		AttrMasking	ROC AUC	0.89	-
		AttentiveEP	ROC AUC	0.85	-
		Uni-Mol	ROC AUC	0.73	-
		GAL 30B	ROC AUC	0.60	30B
		GAL 120B	ROC AUC	0.66	120B
		GAL 6.7B	ROC AUC	0.53	6.7B
		GAL 125 M	ROC AUC	0.39	125 M
		D-MPNN	ROC AUC	0.71	-
		AttentiveFP	ROC AUC	0.66	-
		PretrainGNN	ROC AUC	0.69	-
		Ours	ROC AUC	0.90	8,35,000
**4**	Tox21	BioAct-Het	ROC AUC	0.90	-
		IterRefLSTM	ROC AUC	0.83	-
		Uni-Mol	ROC AUC	0.80	-
		ChemRL-GEM	ROC AUC	0.78	-
		MolXPT	ROC AUC	0.77	350 M
		D-MPNN	ROC AUC	0.76	-
		Grover(Large)	ROC AUC	0.73	More than 100 M
		KA-GCN	ROC AUC	0.79	-
		KA-GAT	ROC AUC	0.79	-
		PretrainGNN	ROC AUC	0.78	-
		Ours	ROC AUC	0.81	8,35,000

For self-supervised contrastive pre-training, we used the ZINC^40^dataset. ZINC is a free database of commercially-available compounds for virtual screening. It contains over 230 million purchasable compounds in ready-to-dock, 3D formats. For our study, we use the 250,000 sampled version. The dataset comprises of 249,455 molecules. We use the benchmark datasets provided in MoleculeNet^15^ for classification. MoleculeNet is a benchmark specially designed for testing Machine Learning(ML) methods of molecular properties. The complete collection comprises of over 700,000 compounds tested on a range of different properties. The benchmark metrics on the MoleculeNet dataset is Area under the Receiver-Operator Curve(ROC-AUC) for classification and Root Mean Square Error(RMSE) for regression. We train and evaluate our proposed model described in Sect. 3 on the following datasets:

- HIV comprises of 41,127 compounds. The task allocated for this dataset is binary classification of whether the given compound has the ability to inhibit HIV replication. The compounds are represented as SMILE strings.
- TOX21 comprises of 7831 compounds. It comprises of qualitative toxicity measurements on 12 biological targets, including nuclear receptors and stress response pathways. The task allocated for this dataset is multi-label classification and the compounds are represented as SMILE strings.
- CLINTOX comprises of 1478 compounds. It comprises of qualitative data of drugs approved by the Food and Drugs Administration(FDA) and those that have failed clinical trials for toxicity reasons. The compounds are represented as SMILE strings.

- BBBP comprises of 2039 compounds. It comprises of binary labels of blood-brain barrier penetration(permeability). The compounds are represented as SMILE strings.

### Performance of contrastive learning

**Fig. 3:**
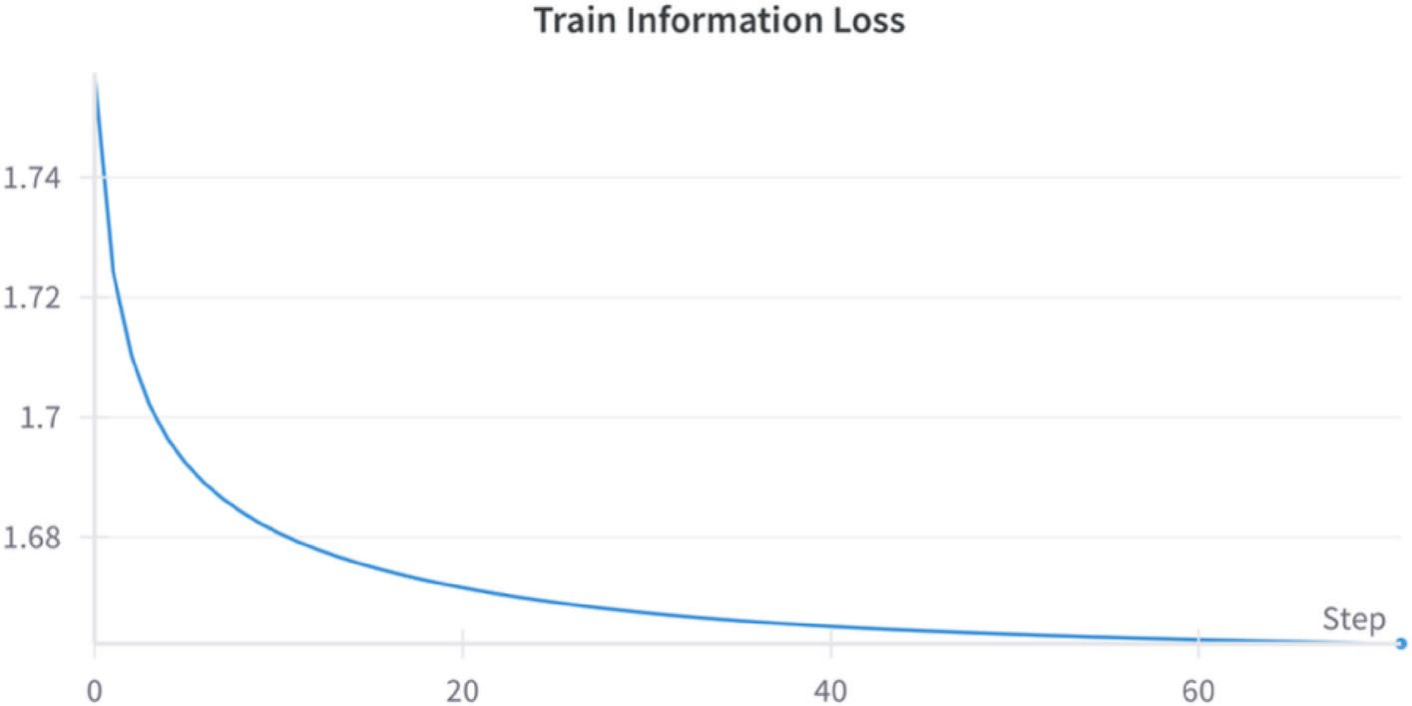
Convergence of Contrastive Learning.

One of the key stages in our proposed approach is the adversarial pre-training of a Graph Convolution(GCN) based encoder network as described in Sect. 3.3.1. We aim to provide a pre-trained knowledge base for the network by learning the distribution of over 240,000 compounds from the ZINC database by a contrastive self-supervised approach. Figure 3 shows the convergence of the training system. From the figure, it is clear that we are able to achieve convergence on the training system despite the potential challenges in training parametric Graph Generative Models. The model is tested using the graph reconstruction loss on the test split which converges to 0.775 after 70 epochs. Since the reconstruction loss is low and optimal on the test split, we can infer that the contrastive system is able to learn a good latent space representation of the graphs and is thereby suitable for knowledge transfer or distillation on downstream classification or regression tasks.

### Experimental validation of base model

In this section, we present the results compared with state of the art benchmarks along with our model’s improved efficiency compared to benchmarks. Table 3compares our models results on the benchmark datasets mentioned in the previous sections. From the table, we can infer that our model achieves state of the art results when compared against benchmarks for all the datasets used in our study along with high parameter efficiency. On the HIV dataset, we achieve an AUC of 79% for only a 835,000 para- metric model outscoring 5 out of the 8 benchmark models. We significantly outscore the Galactica models^10^with a parametric efficiency of over 99%. All Large Language Models have over 50 M parameters with Gal 120B having 120 billion parameters, whereas our approach is trained with only 835,000 parameters thereby achieving faster inference time and better efficiency. The highest score is 81% achieved by^41^, however our proposed model is only 2% away from the highest benchmark despite the significant difference in the number of parameters used for training, the DVMP model used 104.1 million parameters while our approach uses only 835,000 parameters for training and 400,000 parameters for pre-training, thus significantly making our approach more efficient by consistently reducing training and inference time. On Clintox, our proposed model achieves an AUC of 95% outperforming 11 of the 12 benchmarks. We achieve these state of the art results with only 835,000 parameters. The model consistently outperforms all Large language Model based scientific models like Galactica^10^and ChemBerta-2^42^which use over 125 Million and 77 million parameters respectively. Further, our proposed approach is only 0.003% away from the highest benchmark- MolXPT^11^, which uses 350 million parameters thus validating our model on this dataset by both performance and considerable improvement in effi- ciency. For SynFusion^43^, the number of parameters reported is that of the Transformer model used for pre-training introduced in^44^. On BBBP, our proposed model achieves an AUC of 90% outperforming all the benchmarks by a considerable margin. This includes the Galactica models of different sizes in terms of number of parameters, the Grover Large Model^12^which uses over 100 million parameters, ChemBert-a2, Uni-Mol^45^and SPMM^46^. Further, all the models have signficiantly higher number of parameters thus validating our argument on this dataset as well. Finally, on Tox21, we achieve an AUC of 89% outperforming 11 of the 12 benchmarks. Similar to other datasets used in our study, we outperform all the Galactica models, Chembert-a2, ChemRL-GEM, MolXPT and Uni-Mol. These models have over 100 million parameters on average with Galacticas largest model having 130 billion parameters. Further, we also outperform the IterRefLSTM model^47^ which uses one-shot learning. The results obtained on the benchmark datasets validate both the performance and efficiency of our proposed model. On an average, we achieve an AUC of 85% for only 835,000 parameters beating not only Large Language Models(LLMs) but also Graph Neural Network(GNN) baselines. This indicates that our proposed model has the ability to recognize underlying patterns in molecular graphs, effectively embed isomorphic graphs and successfully develop a mapping between the molecular structure and their chemical, biological and physiological properties.

## Attack results

Since our base model has been experimentally validated in the previous section, our attack design’s ability to reduce the model performance validates our strategy. Table 4 shows the reduction in model performance after attack during inference i.e. on the test dataset.

**Table 4:** Evaluation ofmodel performance after proposed attack. Scores are reported after taking an average of 5 test runs.

Dataset	Metric	Normal Conditions	Attack	Decrease %
HIV	AUC	71.7	48.2	22.5
Clintox	AUC	86.9	65.9	21.0
Liphophilicity	MSE	0.677	1.217	44.37
Tox21	AUC	80.8	58.0	22.8

On classification problems, the average performance drop is 23% thereby validating our proposed attack strategy.

## Conclusion

In this study, we devise a technique to effectively predict the properties of molecules thus automating a section in the pipeline of computational drug Discovery. Further, we propose an adversarial attack strategy based on the contrastive embeddings formed during self supervised training. We leverage the pattern recognition abilities of deep learning to model underlying features of molecular graphs. We divided the proposed framework into several changes, namely the initial node and edge featurization, pre-training of a spectral convolution based backbone on the ZINC database that comprises of over 250,000 features and a dual path network that comprises of a spectral and isomorphic branch making the graph neural network expressive and robust. The contrastive self supervised pre-training stage plays a major role in performance and robustness. We pre-train a spectral graph based model to defend it self against adversarial attacks. This is achieved by generating perturbed latent space distributions and negative sampling of edges. The design is trained by penalizing the reconstructed graphs of the perturbed distribution while rewarding the reconstructed graphs of the original latent distribution. Such a setup makes the model aware to adversarial samples which tend to affect the model output for less significant perturbations. Through this design, we achieve a reconstruction loss of only 0.775 on the test split, thus indicating the ability of a contrastive pre-training network to capture underlying features of molecules and effectively compute generate their latent space distributions. We make use of graph isomorphic and spectral branches for our prediction network. The isomorphic network is based on the WL test for graph isomorphism and generates different node embeddings for corresponding nodes in an Automorphic graph. This is significant since in molecules, similar structures may not correspond to similar physical, chemical or biological properties. The spectral branch learns the spectral and spatial underlying features of the molecular graphs. Inter-connections between the 2 branches results in aggregated features within each layer making the model more expressive and robust. Since we achieve positive, convincing results with a more efficient, robust and expressive model, it can pave the way for further research and exploration in using lightweight and efficient models for molecular property prediction. Our proposed model could play a major role in the automation of guiding and testing newly synthesized drugs. Our attack strategy is further validated by the performance on a strong victim model which has been experimentally validated with 5 datasets and over 20 benchmarks models.

## Data Availability

The datasets used in all experiments during this study are available in a publicly accessible repository. They can be obtained at https://pytorch-geometric.readthedocs.io/en/latest/modules/datasets.html. The codes used for all experiments in this study are designed by us and are availabl at https://github.com/Deceptrax123/An-edge-sensitivity-based-gradient-attack-on-GIN-for-inductive-problems
